# Sleep Quality and Sleep Timing in Adolescent Gymnasts: A Cross-Sectional Study

**DOI:** 10.7759/cureus.116922

**Published:** 2026-09-25

**Authors:** Inês Alves Pereira, Beatriz Martins, Joana Branco, Rosário Ferreira

**Affiliations:** 1 Pediatrics, Hospital De Santa Maria, Unidade Local De Saúde Santa Maria, Lisboa, PRT; 2 Pediatrics, Faculdade De Medicina, Universidade De Lisboa, Lisboa, PRT

**Keywords:** adolescent, gymnast athlete, pediatrics, sleep habits, sleep quality, sports

## Abstract

Objectives: Given the limited pediatric-specific evidence in this area, we aimed to examine the association between federated-level gymnastics participation and sleep quality and sleep timing in adolescents. The global Pittsburgh Sleep Quality Index (PSQI) score was defined as the primary outcome, sleep-timing measures as secondary outcomes, and individual PSQI component analyses as exploratory.

Methods: This observational, cross-sectional comparative study included adolescents aged 12 to 16 years participating in federated gymnastics and controls who did not participate in federated sports. Demographic variables included age, sex, and parental educational level, while training-related variables included weekly training duration, number of training sessions, and training schedule. Sleep quality was assessed using the PSQI, and bedtime, wake-up time, sleep duration, and sleep latency were derived from the corresponding PSQI items. Multiple linear regression models included age, sex, maternal educational level, and paternal educational level as covariates.

Results: The study included 60 gymnasts and 37 controls, with baseline differences in age, sex distribution, and parental educational level between groups. Gymnasts trained a median of 12.0 hours/week (Q1-Q3: 8.25 to 20.0) over five sessions/week (Q1-Q3: 4 to 6), with a median training-session end time of 20:30 (range: 18:00 to 22:00). In adjusted analyses, gymnast status was associated with later bedtime (B=0.392 hours, 95% CI 0.043 to 0.740, p=0.028), whereas no statistically significant association was observed between study group and global PSQI score (B=0.311, 95% CI -0.720 to 1.343, p=0.550). Increasing age was associated with later bedtime (B=0.231 hours, p=0.004) and shorter sleep duration (B=-0.398 hours, 95% CI -0.560 to -0.236, *p*<0.001). Among gymnasts, training-session end time was not associated with global PSQI score (B=0.141, 95% CI -0.344 to 0.625, p=0.563; R²=0.006). Overall, 19/97 participants (19.6%) met the conventional PSQI >5 threshold for poor sleep quality, although an optimal adolescent-specific clinical cutoff has not been definitively established.

Conclusion: No statistically significant difference in adjusted global PSQI score was detected between federated adolescent gymnasts and controls. Gymnast status was associated with later bedtime. Approximately one in five participants met the conventional PSQI >5 threshold for poor sleep quality. Given the cross-sectional design, these findings should be interpreted as associations, and larger longitudinal studies incorporating objective sleep measures are warranted.

## Introduction

Sleep is fundamental to physical and psychological well-being and plays an important role in normal growth and development in children. Sleep deprivation and sleep disorders are associated with adverse health outcomes and poorer quality of life. Sleep problems can affect physical health and interfere with cognitive and social functioning [1-3]. Poor sleep is also associated with an increased risk of accidents [4,5].

Longer sleep duration is recommended for adolescents (8 to 10 hours for those aged 14 to 17 years) than for adults (7 to 8 hours); however, adolescents appear to be particularly vulnerable to insufficient sleep because of a combination of physiological and lifestyle factors [6]. Adolescence is a particularly susceptible period for sleep disturbances owing to both biological and environmental factors. Biologically, adolescents experience a delay in circadian phase preference, leading to later bedtimes and subsequently later wake-up times. However, because they are often required to attend school or fulfill other social and family commitments in the morning, later wake-up times are frequently not possible, resulting in shorter sleep duration [6,7]. External factors contributing to later bedtimes include caffeine consumption, schoolwork, extracurricular activities, social engagements, and screen use. In addition to insufficient sleep duration, impaired sleep quality and sleep disturbances are also common among adolescents [8,9].

Regular physical exercise, as opposed to a sedentary lifestyle, is generally associated with better sleep [10,11]. Nonetheless, athletes engaged in high-intensity and high-volume training may exhibit reduced sleep duration and impaired sleep quality, with reported prevalence rates of poor sleep ranging from 28% to 41% [12-14]. However, most studies have focused on adults, while the available evidence on adolescent athletes frequently derives from studies including broad age ranges extending into young adulthood (15 to 26 years). Furthermore, findings have been inconsistent, and it remains unclear whether adolescent athletes have poorer sleep than their non-athlete counterparts.

In addition to school, social, and family commitments, young athletes face the additional demands of training and competition. High training intensity and volume may further affect sleep duration and quality, placing this population at increased risk of insufficient or impaired sleep [15,16].

In addition to school, social, and family commitments, young athletes face the additional demands of training and competition. High training intensity and volume may further affect sleep duration and quality, placing this population at increased risk of insufficient or impaired sleep [15,17]. Litwic-Kaminska and Kotyśko [17], in a study of 335 students comprising athletes and non-athletes, also found that sleep quality was influenced by individual chronotype. A preference for morning activities was associated with better sleep quality, and this relationship was mediated by perceived stress.

Studies focusing specifically on pediatric populations and the association between high-intensity, high-volume exercise and sleep quality are scarce, particularly those directly comparing adolescent athletes with controls. Therefore, this study aimed to examine the association between participation in federated gymnastics and sleep quality and sleep timing in adolescents. The global PSQI score was defined as the primary outcome, sleep-timing measures as secondary outcomes, and individual PSQI component analyses as exploratory. By focusing exclusively on adolescents aged 12 to 16 years participating in federated gymnastics, this study addresses a gap in the available pediatric literature on sleep in young athletes.

## Materials and methods

This was an observational, cross-sectional comparative study of sleep quality in adolescent gymnasts and controls. Participants were recruited using convenience sampling. Participating institutions were approached during the final quarter of 2023, and recruitment and data collection were planned to take place between January and December 2024. Gymnasts were recruited between May and November 2024 through the Portuguese National Gymnastics Federation, affiliated clubs and coaches, and gymnastics events. Of the 60 gymnasts included in the final analysis, 38 completed the questionnaire at a gymnastics competition held on May 25, 2024. Completion of the questionnaire at the event did not necessarily indicate participation in the competition, as some participants attended the event as spectators. Individual participation in the competition on that day was not systematically recorded.

Control participants were recruited through the school boards and teachers of two convenience-selected secondary schools in the Lisbon metropolitan area, Portugal: Agrupamento de Escolas Elias Garcia (Escola Elias Garcia da Sobreda) and Escola Secundária José Afonso, with data collection occurring in January, October, and December 2024. A formal record of the total number of potentially eligible individuals approached or invited to participate was not maintained; therefore, a participation response rate could not be calculated. The timing of questionnaire completion relative to school examinations and other competition periods, apart from the identified gymnastics competition, was not systematically recorded. Informed consent was obtained from all participants' parents or legal guardians, and assent was obtained from the participants when appropriate. Participant initials were used only during data collection to facilitate correct participant identification and prevent questionnaire misattribution; they were not entered into the analytical database. Each participant was assigned a unique study-specific identification code, and all analyses were performed using a de-identified dataset containing no directly identifying information.

The study group included federated gymnasts aged 12 to 16 years. The control group comprised adolescents in the same age range who did not participate in federated sports. Exclusion criteria for both groups included a previous diagnosis of a sleep disorder or asthma or the use of medications commonly associated with sleep disturbances, such as methylphenidate or antihistamines. Asthma was excluded because asthma-related symptoms may independently affect sleep quality, potentially confounding the association under investigation. Eligibility with respect to the medical and medication-related exclusion criteria was determined from the medical and medication history reported in the study questionnaire. No independent verification against medical records was performed.

Data were collected using a two-part questionnaire. The first part gathered demographic information, medical and medication history, and training-related characteristics, including weekly training duration, number of training sessions, and training schedule. Participants in the control group were also asked about their regular physical activity. Parental educational level, categorized as higher education (bachelor’s degree or above) versus lower educational attainment, was included as a demographic variable and considered a proxy for socioeconomic status. Its inclusion was also supported by previously reported associations between parental education and sleep duration and quality in children and adolescents [18,19].

The second part assessed sleep quality using the well-validated Pittsburgh Sleep Quality Index (PSQI), translated and validated for the Portuguese population [20,21]. The global PSQI score is calculated by summing the scores of seven components: subjective sleep quality, sleep latency, sleep duration, habitual sleep efficiency, sleep disturbances, use of sleep medication, and daytime dysfunction. Each component is scored from 0 to 3. The global PSQI score ranges from 0 to 21, with higher scores indicating poorer sleep quality. A score >5 was used to define poor sleep quality, according to the conventional threshold proposed for the PSQI and commonly applied in adolescent populations, although an optimal adolescent-specific clinical cutoff has not been definitively established [8,16,17,20,22]. Bedtime, wake-up time, sleep duration, and sleep latency were derived from the corresponding PSQI items and were also analyzed individually as sleep-timing measures. The global PSQI score was defined as the primary outcome. Sleep-timing metrics were considered secondary outcomes, while analyses of individual PSQI component scores were exploratory.

An a priori sample-size calculation was performed based on the primary outcome, the global PSQI score. Assuming a two-sided significance level of 0.05, 80% statistical power, and an expected standardized between-group difference of Cohen’s d=0.50, a minimum sample size of 64 participants per group was estimated.

Descriptive statistics were used to summarize demographic and clinical characteristics, while comparative analyses were performed to evaluate differences in sleep quality and other potential modulators between the study and control groups. Categorical variables were compared using the chi-square test. For continuous variables, normally distributed data were summarized as mean ± standard deviation (SD) and compared using the independent-samples t-test, whereas non-normally distributed data were summarized as median (25th to 75th percentiles) and compared using the Mann-Whitney U test.

Multiple linear regression analyses were performed to compare global PSQI score, sleep metrics (bedtime, wake-up time, sleep duration, and sleep latency), and individual PSQI component scores between groups, adjusting for potential confounders. Age, sex, maternal educational level, and paternal educational level were included as covariates in the adjusted models because they were considered potential demographic confounders of the association between study group and sleep outcomes. Age and sex are relevant determinants of sleep characteristics during adolescence, while parental educational level was considered a proxy for socioeconomic status. These variables also differed between the study and control groups at baseline and were therefore included as covariates in the multivariable models to account for measured baseline differences. For regression analyses, age was entered as a continuous variable; sex was coded as 0=female and 1=male; study group was coded as 0=control and 1=gymnast; and parental educational level was coded as 1=lower educational attainment and 2=higher education (bachelor’s degree or above).

Among control participants, information on sports participation, number of training sessions per week, weekly training duration, and training schedule was collected. Seventeen of 37 controls (45.9%) reported participation in non-federated sports. Given this substantial heterogeneity in physical activity within the control group, a sensitivity analysis restricting the control group to participants who reported no sports participation was performed to assess the robustness of the primary between-group comparisons.

Assumptions of multiple linear regression were assessed by visual inspection of standardized residual-versus-predicted-value plots for linearity and homoscedasticity, histograms and normal P-P plots of standardized residuals for approximate normality, and tolerance and variance inflation factor values for multicollinearity. Potentially influential observations were assessed using Cook’s distance. Where regression diagnostics indicated substantial deviation from model assumptions, sensitivity analyses using an appropriate transformation of the outcome were performed. Sleep latency was log-transformed as ln(sleep latency + 1) because of marked right-skewing.

Multivariable logistic regression analysis was used to identify factors associated with poor sleep quality (defined according to the conventional PSQI >5 threshold). Given the limited number of poor-sleeper events relative to the number of covariates, this analysis was considered exploratory. Simple linear regression analysis was performed to assess the association between training-session end time and global PSQI score. In the logistic regression model, categorical predictors were entered using indicator coding, with male sex, control status, and higher maternal and paternal educational attainment as the reference categories. For participants who selected one of the predefined training-schedule categories, training-session end time was defined as the upper limit of the selected interval (e.g., 16:00 to 19:00 was coded as 19:00 and 19:00 to 22:00 as 22:00). When participants selected “Other” and provided a specific training schedule, the reported end time was used. Clock-time variables (bedtime, wake-up time, and training-session end time) were converted to decimal hours for regression analyses. For bedtime, times after midnight were coded sequentially beyond 24:00 (e.g., 00:30=24.5) to preserve chronological ordering. Analyses of individual PSQI components were considered exploratory; therefore, no adjustment for multiple comparisons was applied.

There were no missing data for the variables included in the main regression analyses; therefore, no imputation was performed. A p-value <0.05 was considered statistically significant. Statistical analyses were performed using IBM SPSS Statistics, version 26 (IBM Corp., Armonk, NY, USA). The study was reported in accordance with the Strengthening the Reporting of Observational Studies in Epidemiology (STROBE) guidelines for cross-sectional studies. The study was approved by the Ethical Committee of the Lisbon Medical Academic Centre (CAML) (Internal Reference No. 337/22).

## Results

A total of 66 gymnasts and 69 potential controls entered the recruitment and screening process. Six gymnasts were excluded: four because of asthma and two because of regular use of medications potentially interfering with sleep, resulting in 60 gymnasts included in the final analysis. Among potential controls, 24 were excluded because they participated in federated sports and eight because of predefined medical or medication-related exclusion criteria: three because of regular use of medications potentially interfering with sleep, two because of a sleep disorder, two because of asthma, and one because of both asthma and medication use potentially interfering with sleep. The final control group therefore comprised 37 participants. The participant selection process is shown in the flow diagram (Figure 1).

**Figure 1:**
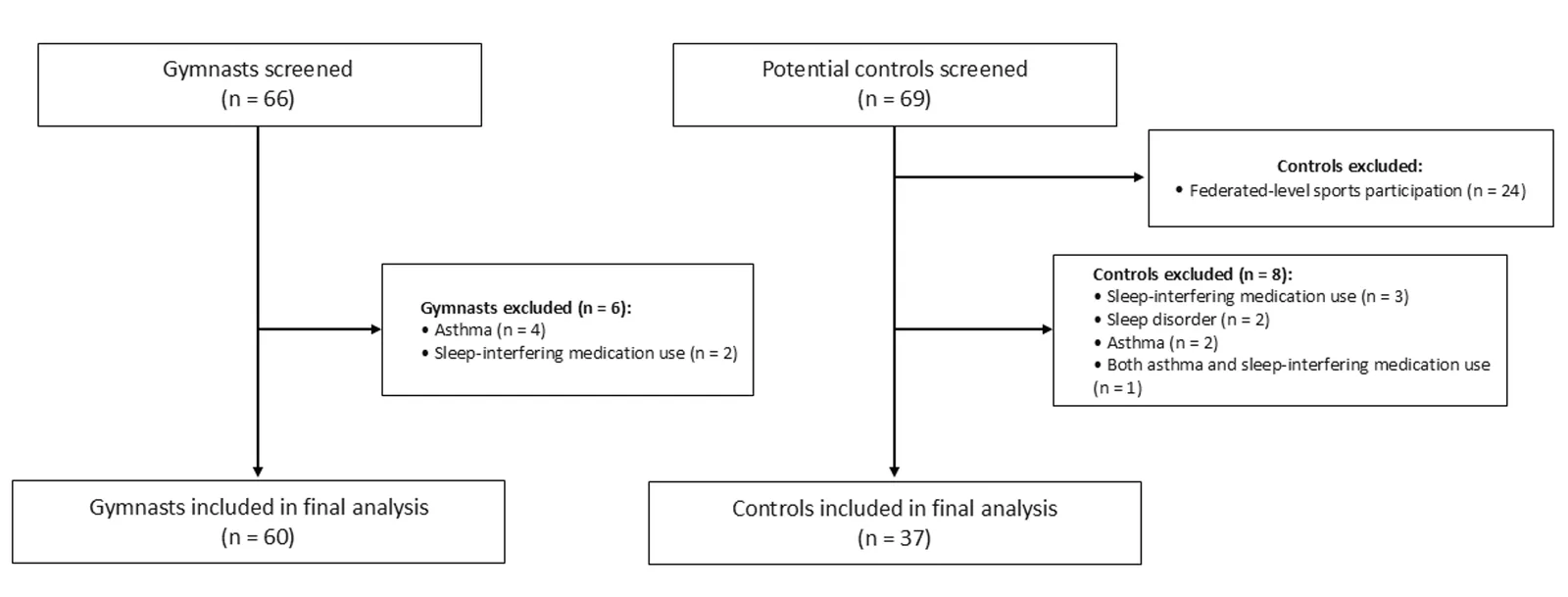
Flow diagram of participant screening, exclusions, and final inclusion in the study

Participant characteristics are presented in Table 1. Gymnasts were older than controls (median age, 14 vs. 13 years), included a higher proportion of female participants (73.3% vs. 43.2%), and more frequently had parents with higher educational attainment (bachelor’s degree or above). As expected, gymnasts reported a significantly higher number of weekly training sessions than controls (median (Q1-Q3): five (4 to 6) vs. 0 (0 to 2), p<0.001). Weekly training duration was also significantly greater among gymnasts (12.0 (8.25 to 20.0) vs. 0 (0 to 2) hours/week, p<0.001). The median training-session end time among gymnasts was 20:30 (range: 18:00 to 22:00). Among controls, 17/37 (45.9%) reported participation in non-federated sports. Among these participants, the median number of weekly training sessions was 2 (Q1-Q3: 2 to 3), corresponding to a median training duration of two hours/week (Q1-Q3: 2 to 3.5). Exercise intensity was not assessed.

**Table 1:** Demographic and sleep characteristics of participants by group ^a ^Mann-Whitney U. ^b ^Chi-squared. ^c ^Independent t-test. ^* ^P-values <0.05 were considered statistically significant. PSQI, Pittsburgh Sleep Quality Index; Q, quartile; SD, standard deviation

	Gymnasts (n=60)	Control (n=37)	P-value^*^
Age: years, median (min-max)	14 (12-16)	13 (12-15)	<0.001^a^
Female: n (%)	44 (73.3)	16 (43.2)	0.003^b^
School year, median (min-max)	9 (6-11)	8 (7-10)	0.056^a^
Mother education level - higher education: n (%)	44 (73.3)	19 (51.4)	0.028^b^
Father education level - higher education: n (%)	37 (61.7)	12 (32.4)	0.005^b^
Training sessions: n, median (Q1-Q3)	5 (4-6)	0 (0-2)	<0.001^a^
Weekly training load: hours, median (Q1-Q3)	12.0 (8.25-20.0)	0 (0-2)	<0.001^a^
Bedtime, median (min-max)	23:00 (21:00-01:00)	22:25 (21:00-00:30)	<0.001^a^
Wake-up time, median (min-max)	7:15 (06:00-08:15)	7:00 (06:00-11:30)	0.246^a^
Hours of sleep, mean (SD)	7.8 (0.81)	8.4 (0.9)	0.02^c^
Time to fall asleep, minutes, median (min-max)	12.5 (1-120)	10 (5-120)	0.65^a^
PSQI score, median (min-max)	4 (0-13)	3 (0-9)	0.03^a^
Poor sleep quality (PSQI >5), n (%)	13 (21.7)	6 (16.2)	0.511^b^

Multiple linear regression analysis was performed to evaluate the association between study group and global PSQI score, adjusting for age, sex, and parental educational level (Table 2). No statistically significant association was found between study group and global PSQI score (B=0.311, 95% CI -0.720 to 1.343, p=0.550). Regression diagnostics identified one potentially influential observation (standardized residual=4.24; Cook’s distance=0.220). The recorded values were verified, and the observation was retained in the primary analysis. A sensitivity analysis excluding this case did not alter the conclusion, with no statistically significant association between study group and global PSQI score (B=-0.006, 95% CI -0.936 to 0.924, p=0.990).

**Table 2:** Effect of study group on sleep characteristics ^* ^P-values of <0.05 were considered statistically significant. Statistical test used: multiple linear regression. B represents the adjusted regression coefficient comparing gymnasts with controls, adjusted for age, sex, maternal educational level, and paternal educational level. Coefficients for bedtime, wake-up time, and sleep duration are expressed in decimal hours. CI, confidence intervals; n, number of subjects; PSQI, Pittsburgh Sleep Quality Index

	B (gymnasts)	95% CI	P-value^*^
Bedtime (hours)	0.392	0.043 to 0.740	0.028
Wake-up time (hours)	0.116	-0.266 to 0.497	0.549
Hours of sleep (hours)	-0.142	-0.503 to 0.218	0.435
Time to fall asleep (minutes)	-3.236	-13.133 to 6.660	0.518
PSQI (score)	0.311	-0.720 to 1.343	0.550

Multiple linear regression analysis was also used to evaluate the association between study group and sleep-related metrics, including bedtime, sleep duration, sleep latency, and wake-up time, adjusting for the same covariates (Table 2). Study group was independently associated with later bedtime (B=0.392 hours, 95% CI 0.043 to 0.740, p=0.028), corresponding to an adjusted difference of approximately 24 minutes later among gymnasts than controls. No statistically significant association was observed between study group and sleep duration (B=-0.142 hours, p=0.435), sleep latency (B=-3.236 minutes, p=0.518), or wake-up time (B=0.116 hours, p=0.549). In the same adjusted models, increasing age was associated with later bedtime (B=0.231 hours per year, p=0.004), corresponding to approximately 14 minutes later bedtime for each additional year of age, and with shorter sleep duration (B=-0.398 hours per year, 95% CI -0.560 to -0.236, p<0.001), corresponding to approximately 24 minutes less sleep per additional year of age. Male sex was associated with longer sleep duration (B=0.400 hours, 95% CI 0.074 to 0.725, p=0.017), corresponding to approximately 24 minutes more sleep among males than females.

In a post hoc sensitivity analysis restricting the control group to participants who reported no sports participation (n=80; 60 gymnasts and 20 controls), no statistically significant association between study group and global PSQI score was observed (B=0.623, 95% CI -0.752 to 1.997, p=0.370). The association with later bedtime was also observed in the restricted analysis and was of greater magnitude (B=0.625 hours, 95% CI 0.180 to 1.070, p=0.007). In contrast to the primary analysis, gymnasts had a significantly shorter adjusted sleep duration than controls reporting no sports participation (B=-0.500 hours, 95% CI -0.950 to -0.050, p=0.030). No statistically significant between-group differences were observed for wake-up time (B=-0.098 hours, 95% CI -0.587 to 0.392, p=0.692) or sleep latency (B=-8.622 minutes, 95% CI -22.304 to 5.061, p=0.213) in the restricted analysis.

One marked outlying observation was identified in the wake-up time model. A sensitivity analysis excluding this observation yielded the same overall conclusion, with no statistically significant association between study group and wake-up time (B=0.166 hours, 95% CI -0.154 to 0.487, p=0.305). One observation with a standardized residual >3 was identified in the sleep-duration model. The recorded values were verified, and the observation was retained in the primary analysis. A sensitivity analysis excluding this case yielded similar estimates and did not alter the conclusions: study group remained not statistically significant (B=-0.112 hours, 95% CI -0.453 to 0.229, p=0.514), while increasing age remained associated with shorter sleep duration (B=-0.391 hours, 95% CI -0.543 to -0.238, p<0.001), and male sex remained associated with longer sleep duration (B=0.349 hours, 95% CI 0.041 to 0.658, p=0.027). The sleep-latency model showed substantial right-skewing of residuals. A sensitivity analysis using log-transformed sleep latency yielded the same overall conclusion, with no statistically significant association between study group and sleep latency (B=-0.229, 95% CI -0.605 to 0.147, p=0.229).

An exploratory multivariable logistic regression analysis was performed to evaluate factors associated with poor sleep quality, defined using the conventional PSQI >5 threshold, adjusting for age, sex, parental educational level, and study group. A total of 19/97 participants (19.6%) met this threshold and constituted the events included in the model. In this exploratory analysis, female sex was associated with higher estimated odds of poor sleep quality (OR=4.19, 95% CI 1.05 to 16.66, p=0.042); however, the wide confidence interval and limited number of events indicate substantial uncertainty and imprecision in this estimate. No statistically significant association was observed between study group and poor sleep quality (gymnasts vs. controls: OR=0.74, 95% CI 0.21 to 2.61, p=0.639). Among gymnasts (n=60), training-session end time was not associated with global PSQI score (B=0.141, 95% CI -0.344 to 0.625, p=0.563; R²=0.006). Regression diagnostics identified one potentially influential observation; a sensitivity analysis excluding this observation yielded the same conclusion (B=-0.011, 95% CI -0.432 to 0.411, p=0.960).

The distribution of individual PSQI component scores was examined in the overall sample and by study group (Table 3). In unadjusted comparisons, the distribution of sleep-disturbance scores differed between groups, with higher ranks among gymnasts than controls (mean rank 52.74 vs. 42.93; Mann-Whitney U=885.5, p=0.020). A similar difference was observed for daytime-dysfunction scores (mean rank 54.31 vs. 40.39; U=791.5, p=0.010). No statistically significant between-group differences were observed for the remaining PSQI components. Among gymnasts, 53/60 (88%) had a non-zero sleep-disturbance component score, and 43/60 (72%) had a non-zero daytime-dysfunction component score.

**Table 3:** PSQI component scores by study group ^* ^P-values <0.05 were considered statistically significant. Comparative statistics test used: Mann-Whitney U test. PSQI, Pittsburgh Sleep Quality Index; Q1, first quartile; Q3, third quartile

	Score, median (Q1-Q3)			
	All participants	Gymnasts (n=60)	Control (n=37)	P-value^*^
1. Subjective sleep quality	1 (0-1)	1 (1-1)	1 (0-1)	0.175
2. Sleep latency	1 (0-1)	1 (0-1)	1 (0-1)	0.617
3. Sleep duration	0 (0-0)	0 (0-1)	0 (0-0)	0.166
4. Habitual sleep efficiency	0 (0-0)	0 (0-0)	0 (0-0)	0.888
5. Sleep disturbances	1 (1-1)	1 (1-1)	1 (0-1)	0.020
6. Use of sleeping medication	0 (0-0)	0 (0-0)	0 (0-0)	0.131
7. Daytime dysfunction	1 (0-1)	1 (0-1)	1 (0-1)	0.010

In the adjusted exploratory analysis of individual PSQI components (Table 4), gymnast status was associated with a higher sleep-disturbance score (B=0.271, 95% CI 0.040 to 0.503, p=0.022), while no statistically significant associations were observed for the remaining PSQI components. Because no adjustment for multiple comparisons was applied, all component-level findings should be interpreted as exploratory.

**Table 4:** Effect of group of study on PSQI component scores ^* ^P-values of <0.05 were considered statistically significant. Statistical test used: multiple linear regression; B represents the adjusted regression coefficient comparing gymnasts with controls, adjusted for age, sex, maternal educational level, and paternal educational level. CI, confidence intervals; n, number of subjects; PSQI, Pittsburgh Sleep Quality Index

	B (gymnasts)	95% CI	P-value^*^
1. Subjective sleep quality	0.105	-0.149 to 0.359	0.413
2. Sleep latency	-0.030	-0.460 to 0.400	0.890
3. Sleep duration	-0.100	-0.295 to 0.096	0.315
4. Habitual sleep efficiency	-0.036	-0.226 to 0.153	0.705
5. Sleep disturbances	0.271	0.040 to 0.503	0.022
6. Use of sleeping medication	-0.128	-0.316 to 0.060	0.180
7. Daytime dysfunction	0.229	-0.129 to 0.587	0.208

## Discussion

Our study compared several sleep-related measures between adolescents participating in federated gymnastics and controls to better understand the association between participation in federated gymnastics and sleep quality and timing.

In multivariable models including age, sex, and parental educational level as covariates, gymnast status was associated with later bedtime compared with controls, while sleep duration was numerically shorter among gymnasts but did not differ significantly between groups. The literature is conflicting regarding sleep duration, with two studies using self-reported data in young people aged 15 to 23 years reporting that athletes, specifically football players, obtained more sleep [23,24]. In contrast, a study using objective sleep duration data reported shorter sleep duration in athletes than in non-athletes [25]. In the general population, sleep durations below eight hours have been associated with poorer cognitive performance, including reaction time and cognition, as well as impaired mood, reduced alertness, and increased daytime sleepiness [26]. This becomes even more relevant in the athletic setting, as sleep is crucial for preparation for and recovery from training and competition and has been associated with better performance outcomes in young athletes [6,27,28]. The mean sleep duration among gymnasts in this study was 7.8 hours. Although athletic performance and injury occurrence were not assessed in this study, previous studies have associated sleep durations below eight hours with poorer performance-related outcomes and an increased risk of musculoskeletal injury in young athletes [28-30]. As expected, age was independently associated with later bedtimes and shorter sleep duration, consistent with the well-established tendency for older adolescents to have later bedtimes and shorter sleep duration.

Regarding sleep latency, no statistically significant between-group difference was observed. The available evidence is also conflicting regarding this sleep parameter: in one study, football players reported longer sleep latency than non-athletes, whereas in another, athletes reported shorter sleep latency [23,24].

Although the unadjusted global PSQI score was higher among gymnasts, this difference was attenuated after adjustment for age, sex, and parental educational level, and no statistically significant adjusted difference was detected between groups. The planned sample size was not achieved, particularly in the control group. With the achieved sample sizes of 60 gymnasts and 37 controls, the study had approximately 66% power to detect the prespecified standardized between-group difference of Cohen’s d=0.50, compared with the 80% power originally targeted. Therefore, the non-significant primary finding should not be interpreted as evidence of equivalence between groups. No statistically significant between-group difference was observed in the proportion or adjusted odds of poor sleep quality. These findings differ from those of some previous studies in young athletes, which reported poor-sleep prevalence rates of 32% to 42%, sleep duration below seven hours in 39%, and daytime sleepiness in 51% of participants [16,31]. The multivariable logistic regression analysis of poor sleep quality was based on only 19 events. Given the number of covariates included in the model, the analysis may be susceptible to overfitting and limited statistical precision and should therefore be regarded as exploratory. In the same model, female sex showed higher estimated odds of poor sleep quality; however, the small number of poor-sleeper events and wide confidence interval indicate substantial uncertainty around this estimate, which should therefore be interpreted cautiously.

To explore which specific dimensions of sleep might differ between groups, we examined the individual PSQI components. In unadjusted comparisons, the distributions of sleep-disturbance and daytime-dysfunction scores differed between groups, with higher ranks among gymnasts. After adjustment for age, sex, and parental educational level, the sleep-disturbance component was the only component with a p-value <0.05, whereas no statistically significant associations were observed for the remaining PSQI components, including daytime dysfunction. As seven PSQI components were examined, a Bonferroni-corrected significance threshold would be approximately 0.007 (0.05/7). None of the component-level findings met this threshold, including the unadjusted differences in sleep disturbance (p=0.020) and daytime dysfunction (p=0.010), and the adjusted association with sleep-disturbance score (p=0.022). These findings should therefore be regarded as exploratory and interpreted cautiously.

Late bedtimes and shorter sleep duration may be associated with several factors, including late training sessions, competition schedules, academic demands, and increased physiological arousal after exercise. In this study, most athletes trained in the late afternoon or evening, with a median training end time of 20:30 and some sessions ending as late as 22:00. However, among gymnasts, training-session end time was not associated with global PSQI score (R²=0.006, p=0.563). Given the heterogeneity in physical activity within the control group, we performed a post hoc sensitivity analysis restricting the comparison to controls reporting no sports participation. No association with global PSQI score was observed in this restricted analysis, while the association with later bedtime was also observed. In contrast, shorter sleep duration was observed among gymnasts in the restricted analysis. Because this post hoc analysis included only 20 inactive controls, this finding is imprecise and should not be interpreted as establishing a difference in sleep duration between gymnasts and inactive controls. It nevertheless raises the possibility that physical-activity heterogeneity within the full control group may have influenced the between-group comparison of sleep duration.

Previous studies suggest that training and competition timing may be relevant to sleep patterns in young athletes. A study by Heishmann et al. [32] concluded that morning training sessions were associated with shorter sleep duration compared with afternoon sessions (13:45 to 16:00). Additionally, Fox et al. [6] reported later sleep onset and shorter sleep duration following evening competitions (19:10 start time) compared with daytime competitions (15:45). Taken together, these findings from previous studies suggest that the timing of training and competition may be associated with sleep duration and should be considered when evaluating sleep patterns in young athletes, but this remains a hypothesis requiring prospective investigation in our population.

A total of 19/97 participants (19.6%) met the conventional PSQI >5 threshold for poor sleep quality, although an optimal adolescent-specific clinical cutoff has not been definitively established, indicating that sleep-quality concerns were present in both groups. The determinants of this finding cannot be established from the variables assessed in the present study, but it highlights the broader public health implications regarding sleep quality beyond the athletic context. The prevalence of poor sleep quality in our sample (19/97, 19.6%) is consistent with evidence that sleep problems are common among Portuguese adolescents. In a large study of 6,919 Portuguese secondary-school students, 21.4% reported insomnia symptoms, and 29.3% had insufficient sleep (<8 h/night) [33]. More recent national HBSC data also indicate frequent sleep difficulties among Portuguese adolescents [34]. However, direct comparison should be made cautiously because these studies used different definitions and instruments to assess sleep problems.

This study has several limitations. First, sleep outcomes were assessed using self-reported questionnaires, and no objective sleep measures, such as actigraphy or polysomnography, were used. Therefore, estimates of sleep duration, timing, and quality may be subject to recall and reporting bias and may not fully reflect objectively measured sleep.

Although an a priori sample-size calculation estimated a required sample of 64 participants per group, this target was not achieved, particularly in the control group. Consequently, the study may have had limited power to detect smaller between-group differences, and non-significant findings should not be interpreted as evidence of equivalence between gymnasts and controls. In addition, the multivariable logistic regression analysis of poor sleep quality included only 19 events, corresponding to approximately 3.8 events per covariate. This relatively low events-per-predictor ratio may limit the precision and stability of the estimates.

The different recruitment strategies used for the two groups may also have introduced selection bias, as gymnasts were recruited through the Portuguese National Gymnastics Federation, affiliated clubs, coaches, and gymnastics events, whereas controls were recruited through two convenience-selected secondary schools in the Lisbon metropolitan area. A formal record of the total number of eligible individuals approached or invited to participate was not maintained, precluding calculation of a participation response rate and formal assessment of non-response bias. These recruitment limitations restrict the generalizability of the findings to the broader population of federated adolescent gymnasts. Recruitment through federation- and club-based channels may also have preferentially included more engaged or well-supported gymnasts, although the direction of any resulting selection bias on sleep outcomes cannot be determined. The timing of data collection also differed between groups. Gymnast data were collected between May and November 2024, whereas control-group data were collected in January, October, and December 2024. A substantial proportion of gymnasts completed the questionnaire at a gymnastics competition event; however, attendance at the event did not necessarily indicate participation in the competition, as some participants attended as spectators. Individual competition participation at the time of questionnaire administration was not systematically recorded. Therefore, the potential influence of competition-related training, stress, travel, or other contextual factors on PSQI responses could not be specifically assessed. As the PSQI assesses sleep during the preceding month, seasonal variation, school schedules, examination periods, holidays, and competition-related demands may have influenced reported sleep outcomes. The timing of questionnaire completion relative to other competitions, school examinations, and holidays was not systematically recorded. The non-contemporaneous timing of data collection may have introduced systematic differences in the between-group comparisons. Generalizability is also limited by the inclusion of a single sporting discipline, recruitment within one metropolitan area, and the exclusion of adolescents with asthma.

The study and control groups were not well matched at baseline with regard to age, sex distribution, and parental educational level. In particular, gymnasts were significantly older than controls, and age was independently associated with later bedtime and shorter sleep duration. Although these variables were included in the adjusted analyses, statistical adjustment may not fully account for the baseline differences between groups, particularly given the relatively small sample, and residual confounding cannot be excluded. The age ranges also did not fully overlap, as 16-year-old participants were present only in the gymnast group, further limiting the ability of statistical adjustment to fully address the age imbalance. Parental educational level was used as a proxy for socioeconomic status; however, other socioeconomic indicators, such as household income and parental occupation, were not assessed. Other potentially relevant factors, including chronotype, screen use, caffeine intake, and academic demands, were not systematically assessed and may represent additional sources of residual confounding.

The control group was heterogeneous with regard to physical activity, with 17/37 (45.9%) participants reporting non-federated sports participation. Although exercise frequency and duration were collected, exercise intensity was not assessed. A post hoc sensitivity analysis restricted to controls reporting no sports participation was therefore performed. Because this restricted analysis included only 20 controls, its estimates were less precise and should be interpreted cautiously. The restricted analysis did not materially alter the findings for global PSQI score, bedtime, wake-up time, or sleep latency, although a difference in sleep duration was observed, with gymnasts sleeping approximately 30 minutes less than inactive controls. This finding raises the possibility that heterogeneity in physical activity within the control group may have attenuated the between-group difference in sleep duration observed in the primary analysis.

Finally, multiple PSQI components were examined without adjustment for multiple comparisons; therefore, the possibility of type I error cannot be excluded. Component-level findings should consequently be regarded as exploratory and interpreted cautiously. Further longitudinal studies with larger samples, including athletes from different sports, are needed and should incorporate objective measures of both physical activity and sleep, such as accelerometry or actigraphy and, where appropriate, polysomnography.

## Conclusions

In this study, no statistically significant difference in adjusted global PSQI score was observed between adolescents participating in federated gymnastics and controls. Gymnast status was associated with later bedtime after adjustment for age, sex, and parental educational level. Approximately one in five participants met the conventional PSQI >5 threshold for poor sleep quality, although an optimal adolescent-specific clinical cutoff has not been definitively established. Training-session end time was not associated with global PSQI score among gymnasts.

Given the cross-sectional design, the smaller-than-planned control sample, baseline differences between groups, reliance on self-reported sleep measures, and potential residual confounding, these findings should not be interpreted as evidence of causality or equivalence between groups. Larger longitudinal studies incorporating objective measures of sleep and physical activity, as well as detailed assessment of training timing and other determinants of adolescent sleep, are needed.

## Appendices

Appendix A: questionnaire for study group 

This questionnaire aims to characterize the study participants through questions regarding demographic information, sports participation, and medical history. All information collected is confidential and will be handled in accordance with the study’s data-protection procedures.

Participant Identification

Participant Initials: ____________________ ID #: ____________________ Date: ____ / ____ / ______

Demographic Data

1. Age: ____________________

2. Sex: ____________________

3. School Grade: ____________________

4. Parents’ Educational Level

Mother: ☐ Primary education ☐ Secondary education ☐ Bachelor’s degree ☐ Master’s degree ☐ Doctorate

Father: ☐ Primary education ☐ Secondary education ☐ Bachelor’s degree ☐ Master’s degree ☐ Doctorate

Federated Sports Participation

5. Sport: ____________________

6. Competition Category / Age Group: ____________________

7. Training Load

Number of training sessions per week: ______

Total training hours per week: ______ hours

Usual training schedule:

☐ 9:00-13:00 ☐ 14:00-16:00 ☐ 16:00-19:00 ☐ 19:00-22:00 ☐ Other: ____________________

Additional Sports Participation

8. Do you practice any other sport?

☐ Yes  ☐ No

If Yes, please answer the following questions. If No, proceed to Question 10.

9. Sport: ____________________

10. Training Load

Number of training sessions per week: ______

Total training hours per week: ______ hours

Training schedule: ____________________

Medical History

11. Do you have, or have you ever been diagnosed with a chronic medical condition?

☐ Yes  ☐ No

(e.g., asthma or other respiratory diseases, epilepsy or other neurological disorders, obstructive sleep apnea or other sleep disorders, depression or other psychiatric disorders)

If yes, please specify:______________________________________

12. Do you take any regular or long-term medication?

☐ Yes  ☐ No

If yes, please specify:______________________________________

Appendix B: questionnaire for control group

This questionnaire aims to characterize the study participants through questions regarding demographic information, sports participation, and medical history. All information collected is confidential and will be handled in accordance with the study’s data-protection procedures.

Participant Identification

Participant Initials: ____________________ ID #: ____________________ Date: ____ / ____ / ______

Demographic Data

1. Age: ____________________

2. Sex: ____________________

3. School Grade: ____________________

4. Parents’ Educational Level

Mother: ☐ Primary education ☐ Secondary education ☐ Bachelor’s degree ☐ Master’s degree ☐ Doctorate

Father: ☐ Primary education ☐ Secondary education ☐ Bachelor’s degree ☐ Master’s degree ☐ Doctorate

Sports Participation

5. Do you practice any sport?

☐ Yes  ☐ No

If Yes, please answer the following questions. If No, proceed to Question 9.

6. Sport: ____________________

7. Are you a federated athlete?

☐ Yes  ☐ No

8. Training Load

Number of training sessions per week: ______

Total training hours per week: ______ hours

Training schedule: ____________________

Medical History

9. Do you have, or have you ever been diagnosed with a chronic medical condition?

☐ Yes  ☐ No

(e.g., asthma or other respiratory diseases, epilepsy or other neurological disorders, obstructive sleep apnea or other sleep disorders, depression or other psychiatric disorders)

If yes, please specify:_______________________________________

10. Do you take any regular or long-term medication?

☐ Yes  ☐ No

If yes, please specify:_______________________________________
